# Mesaconine alleviates doxorubicin-triggered cardiotoxicity and heart failure by activating PINK1-dependent cardiac mitophagy

**DOI:** 10.3389/fphar.2023.1118017

**Published:** 2023-04-12

**Authors:** Ji-Chao Zhou, Cai-Cai Jin, Xiao-Li Wei, Rui-Bing Xu, Ruo-Yu Wang, Zhi-Meng Zhang, Bo Tang, Jin-Mei Yu, Jiao-Jiao Yu, Shuang Shang, Xiao-Xi Lv, Fang Hua, Ping-Ping Li, Zhuo-Wei Hu, Yong-Mei Shen, Feng-Peng Wang, Xiu-Ying Ma, Bing Cui, Fu-Neng Geng, Xiao-Wei Zhang

**Affiliations:** ^1^ State Key Laboratory of Bioactive Substance and Function of Natural Medicines, CAMS Key Laboratory of Molecular Mechanism and Target Discovery of Metabolic Disorder and Tumorigenesis, Institute of Materia Medica, Chinese Academy of Medical Sciences & Peking Union Medical College, Beijing, China; ^2^ Sichuan Engineering Research Center for Medicinal Animals, Sichuan, China; ^3^ Department of Chemistry of Medicinal Natural Products, West China College of Pharmacy, Sichuan University, Sichuan, China; ^4^ State Key Laboratory of Biotherapy and Cancer Center, West China Hospital, Sichuan University and Collaborative Innovation Center of Biotherapy, Sichuan, China

**Keywords:** *Aconitum carmichaelii*, mitochondrial homeostasis, myocardial energy metabolism, oxidative stress injury, Sqstm1

## Abstract

Aberrant mitophagy has been identified as a driver for energy metabolism disorder in most cardiac pathological processes. However, finding effective targeted agents and uncovering their precise modulatory mechanisms remain unconquered. Fuzi, the lateral roots of *Aconitum carmichaelii*, shows unique efficacy in reviving Yang for resuscitation, which has been widely used in clinics. As a main cardiotonic component of Fuzi, mesaconine has been proven effective in various cardiomyopathy models. Here, we aimed to define a previously unrevealed cardioprotective mechanism of mesaconine-mediated restoration of obstructive mitophagy. The functional implications of mesaconine were evaluated in doxorubicin (DOX)-induced heart failure models. DOX-treated mice showed characteristic cardiac dysfunction, ectopic myocardial energy disorder, and impaired mitophagy in cardiomyocytes, which could be remarkably reversed by mesaconine. The cardioprotective effect of mesaconine was primarily attributed to its ability to promote the restoration of mitophagy in cardiomyocytes, as evidenced by elevated expression of PINK1, a key mediator of mitophagy induction. Silencing *PINK1* or deactivating mitophagy could completely abolish the protective effects of mesaconine. Together, our findings suggest that the cardioprotective effects of mesaconine appear to be dependent on the activation of PINK1-induced mitophagy and that mesaconine may constitute a promising therapeutic agent for the treatment of heart failure.

## Introduction

Heart failure (HF), a leading cause of death worldwide, is the end-stage consequence of various cardiovascular diseases (Heidenreich et al., 2022). Although numerous signaling pathways and molecular components have been identified in the initiation and progression of HF, the pathogenesis of HF remains incompletely understood (GBD 2019 Risk Factors Collaborators, 2020; Arrigo et al., 2020; He et al., 2022). To date, the clinical treatment for HF mainly focuses on symptom relief, but it is insufficient to significantly prolong survival and improve prognosis in patients with HF (Zannad, 2018; Murphy et al., 2020). Investigating specific therapeutic agents for HF remains an unconquered area in drug development.

Accumulating evidence indicates that myocardial energy metabolism disorder is not only a critical reason for the reduction in systolic force in the early stages of HF, but also an essential trigger for myocardial fibrosis and loss of cardiomyocytes in the late stages (Lopaschuk et al., 2021). Since mitochondria are the major organelles for the energy supply of cardiomyocytes, their homeostasis is important for maintaining cardiac structure and function (Brown et al., 2017). In addition, as the most important mechanism of mitochondrial quality control, mitochondrial autophagy (also termed mitophagy) can promote the turnover of mitochondria and prevent the accumulation of dysfunctional mitochondria (Palikaras et al., 2017). When mitochondria are damaged, their inner membrane becomes depolarized, leading to PINK1 accumulation at the mitochondrial outer membrane, which is necessary for the recruitment and phosphorylation of Parkin at the mitochondrial surface (Saito and Sadoshima, 2015). Sufficient Parkin further ubiquitinates multiple proteins of the outer membrane (Shao et al., 2020; Saito et al., 2021), which are recognized by SQSTM1 (also termed p62), an autophagic substrate receptor involved in the trafficking of impaired mitochondria to the degradation pathway (Saito and Sadoshima, 2015). Numerous studies have indicated that impaired mitophagy is involved in cardiac dysfunction caused by abnormal myocardial energy metabolism (Billia et al., 2011; Ranjbarvaziri et al., 2021). Hence, restoring mitophagy could be a potential therapeutic strategy for the treatment of HF.

Doxorubicin (DOX), an anthracycline antibiotic, has an extensive antitumor effect against assorted solid and hematologic tumors (Carvalho et al., 2009). However, dose cumulative cardiotoxicity is a major limitation for its clinical application (Singal and Iliskovic, 1998; Yeh and Bickford, 2009; Levis et al., 2017). The cardiotoxicity of DOX involves a variety of mechanisms, among which the most accepted cardiotoxic mechanism is the ability of DOX to increase excess mitochondrial-related reactive oxygen species (ROS) production (Ma et al., 2020). The anthraquinone structure of DOX endows it with a high affinity for cardiolipin, a phospholipid exclusively localized at the inner mitochondrial membrane, which is then catalyzed into semiquinone doxorubicin (SQ-DOX) through electron transfer from nicotinamide adenine dinucleotide phosphate (NADPH) oxidase, nitric oxide synthases (NOSs), and the mitochondrial electron transport chain (ETC) (Bachur et al., 1979; Vasquez-Vivar et al., 1997). SQ-DOX is a kind of unstable metabolite that can be oxidized within mitochondria, accompanied by excessive ROS production, which in turn causes a drastic energy metabolism disorder in cardiomyocytes and eventually progresses to HF (Davies and Doroshow, 1986; Mukhopadhyay et al., 2009). Therefore, the DOX-induced cardiotoxicity model is an appropriate model to evaluate the effects of drug candidates for HF treatment and to investigate the mechanisms of mitochondrial dysfunction in HF.

Active ingredients derived from medicinal plants are an essential source for the innovative drug development of HF. Fuzi, the lateral roots of *Aconitum carmichaelii*, is a representative medicine for invigorating Yang and saving lives. It has been widely used as a cardiotonic drug in the clinical applications of traditional Chinese medicine (Liu et al., 2012; Yang et al., 2014; Chen et al., 2022). As a type of aconitine, mesaconine (MES) is an active compound isolated from Fuzi with a broad spectrum effect on biomedical pharmacology (Jian et al., 2012). Our preliminary pharmacodynamics study showed that mesaconine exhibited significant protective effects against DOX-induced cardiac dysfunction and myocardial injury. Additionally, we found that mesaconine efficiently mitigated mitochondrial ROS generation and promoted ATP production in DOX-treated primary cardiomyocytes. However, the specific mechanisms of the cardioprotective effects of mesaconine on HF have not been fully elucidated.

Taken together, we speculated that mesaconine would have therapeutic benefits against cardiac dysfunction by maintaining mitochondrial homeostasis. Indeed, our present study indicates that mesaconine treatment could dramatically promote the expression of PINK1 to restore DOX-induced mitophagy dysfunction, which is essential for the clearance of damaged mitochondria and maintaining the energy homeostasis of cardiomyocytes. Our study reveals a previously unidentified cardioprotective mechanism of mesaconine. It suggests that mesaconine is a novel and attractive therapeutic molecule for myocardial energy metabolism disorder during the development of heart failure.

## Results

### Mesaconine protects cardiomyocytes against DOX-induced cell death and ROS generation

**FIGURE 1:**
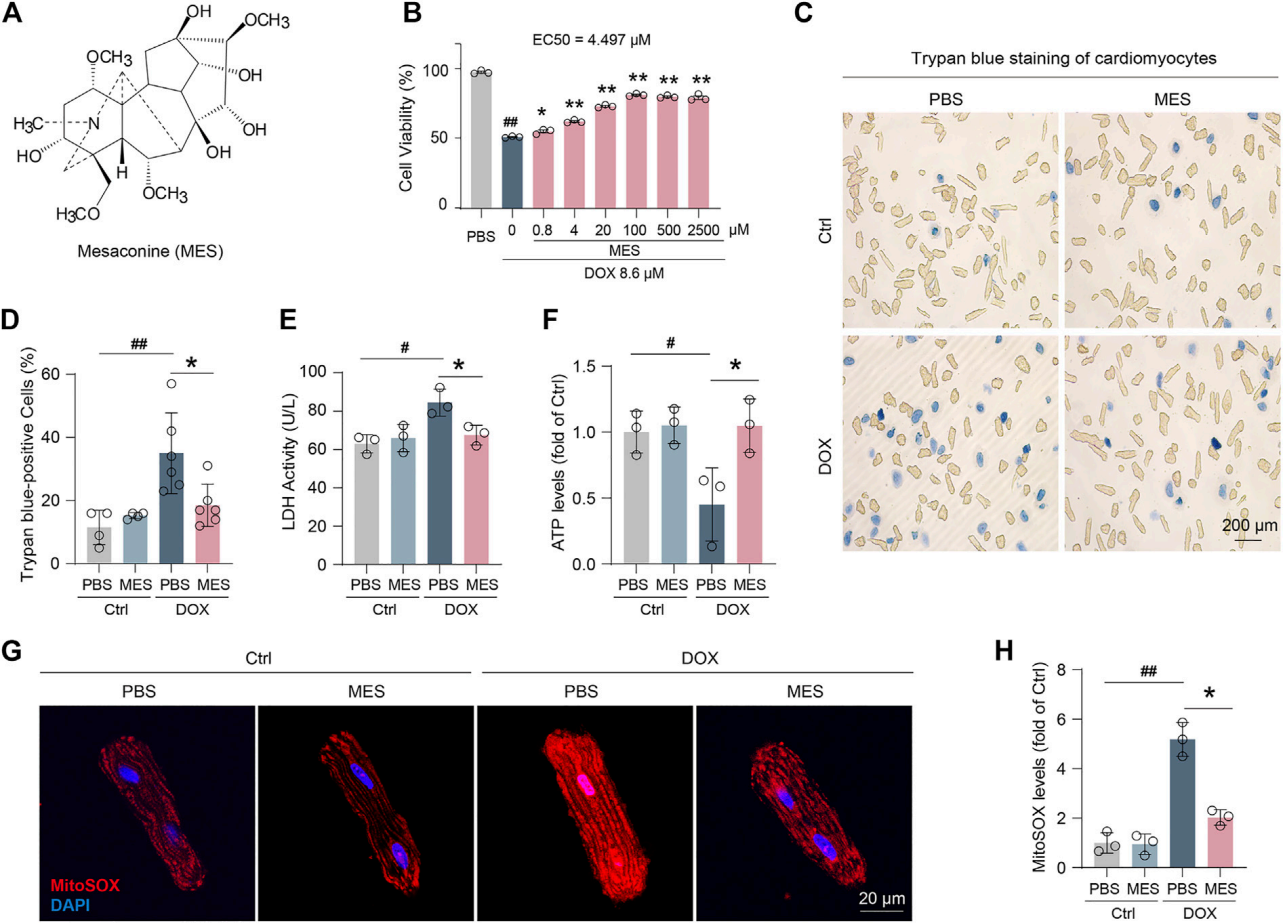
Mesaconine attenuated DOX-induced cardiomyocytes injury. **(A)** Chemical structure of mesaconine. **(B)** The cell viability of DOX and/or mesaconine-treated cardiomyocytes were detected by CCK-8 assay. **(C)** Representative images of cardiomyocytes stained with trypan blue (n = 5). **(D)** Quantitative analysis of trypan blue positive cardiomyocytes. **(E, F)** Treatment mesaconine showed resistance to DOX-induced LDH release **(E)** and reduced ATP production **(F)**. **(G)** Representative images of MitoSOX (mitochondrial ROS) levels were measured by immunofluorescence staining. **(H)** Quantification of MitoSOX levels. Data are represented as means ± SEM of at least 3 biologically independent replicates assays. *, # *p* < 0.05; **, ## *p* < 0.01.

To determine whether mesaconine (Figure 1A) attenuates DOX-induced cardiomyocyte toxicity, we performed a cell viability assay in cultured adult rat ventricular myocytes. Mesaconine could dose-dependently augment cell viability upon DOX treatment, reaching a plateau with concentrations ranging from 100 to 2500 µM (EC50 = 4.497 µM, Figure 1B). After 24 h of treatment with DOX at 8.6 µM, 35% of the cardiomyocytes were not viable, as observed under optical microscopy. However, DOX-induced cell death was markedly diminished in the mesaconine-treated group (Figures 1C, D). Similarly, leakage of lactate dehydrogenase (LDH) upon DOX, a biochemical marker for necrotic cell death, was significantly suppressed following mesaconine treatment (Figure 1E). Since mitochondrial dysfunction is the major cause of energy production disorder in cardiomyocytes, ATP contents were detected to evaluate mitochondrial function. We observed that levels of ATP were dramatically decreased following treatment with DOX, which could be turned over by mesaconine in primary cardiomyocytes (Figure 1F). Moreover, ROS accumulation is the primary biological event during DOX-induced myocardial injury. ROS generation was examined by MitoSOX staining. According to the confocal analysis, we found that DOX treatment increased mitochondrial ROS levels, which could be significantly ameliorated by mesaconine treatment (Figures 1G, H). Collectively, these findings indicate that mesaconine protects cardiomyocytes against DOX-triggered oxidative stress injury.

### Mesaconine ameliorates DOX-induced cardiac dysfunction and remodeling in acute DOX-induced cardiotoxicity mice

**FIGURE 2:**
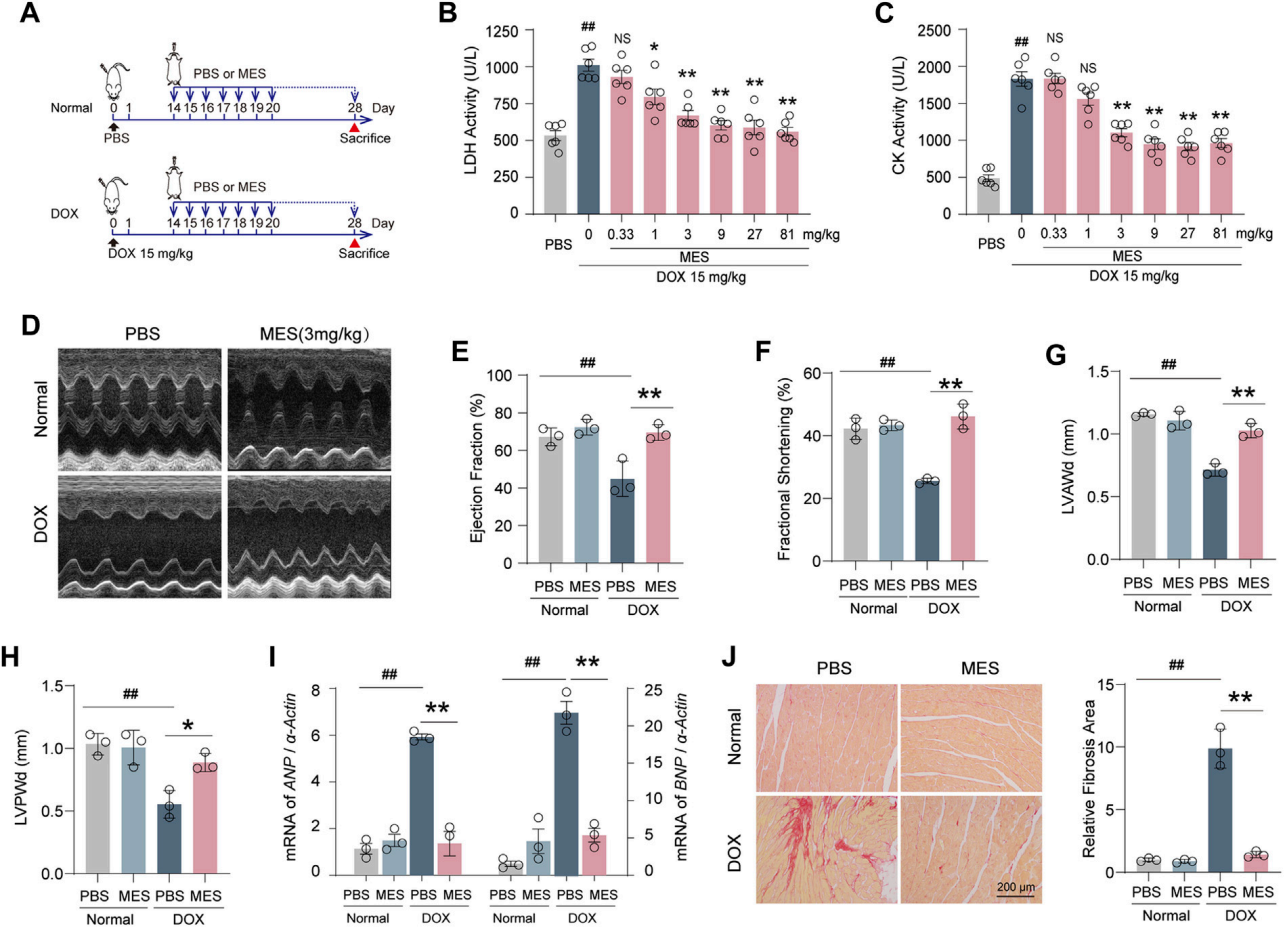
Mesaconine protected mice from acute DIC by reducing cardiac dysfunction and remodeling. **(A)** Flowchart for preparing acute DIC model and corresponding treatments. **(B, C)** The dose-dependent serum activity of LDH and CK. **(D)** Representative images of left ventricular M-mode echocardiograms (n = 6–8/group). Left ventricular ejection fraction [EF, **(E)**], fractional shortening [FS, **(F)**], and diastolic anterior wall thickness [LVAWd, **(G)**], posterior wall thickness [LVPWd, **(H)**] were measured by echocardiography. **(I)** RT-PCR detected mRNA expression of *ANP* and *BNP*. **(J)** Representative images of Sirius red stained heart sections and a summary of collagen deposition. Data are represented as means ± SEM of at least 3 biologically independent replicates assays. * *p* < 0.05; **, ## *p* < 0.01.

To confirm our *in vitro* findings, an acute DOX-induced cardiotoxicity (DIC) mouse model was established to verify whether mesaconine could improve acute myocardial injury induced by DOX (Figure 2A). Then, we treated the DOX injured mice with mesaconine ranging from 0.33 to 81 mg/kg and tested their serum myocardial enzyme activity. Given that the 3 mg/kg dosage was sufficient to significantly improve cardiac function as indicated by the LDH and creatine kinase (CK) levels (Figures 2B, C), we chosen this minimum effective dose for the following *in vivo* experiments. The echocardiography result found that 28 days after the initial treatment with DOX, the mice exhibited visible left ventricular (LV) dysfunction and remodeling, which is consistent with previous reports (Liu et al., 2019) (Figure 2D). While the mesaconine treatment greatly improved DOX-induced cardiac dysfunction and dilation manifested by a higher LV ejection fraction (EF, Figure 2E) and fractional shortening (FS, Figure 2F), as well as thicker diastolic LV anterior wall thickness (LVAWd, Figure 2G) and LV posterior wall thickness (LVPWd, Figure 2H). We next assessed the protective effects of mesaconine against myocardial injury by detecting the relative mRNA expression of atrial natriuretic peptide (*ANP*) and B-type natriuretic peptide (*BNP*), which are the primary diagnostic and predictive markers in heart failure. As expected, DOX treatment promoted a remarkable expression of *ANP* and *BNP* in the heart (by ∼6 times and ∼7 times *vs*. normal hearts, respectively). In contrast, the elevated transcript levels of *ANP* and *BNP* were significantly decreased after mesaconine administration (Figure 2I). In addition, treatment of mice with mesaconine markedly reduced DOX-induced cardiac fibrosis, as indicated by improved myocardial collagen deposition. An 80% reduction in collagen deposition was observed in the heart tissues of mesaconine-treated mice compared with tissues from DOX mice (Figure 2J). These data provide support for the myocardial protective effects of mesaconine *in vivo*.

### Mesaconine attenuates myocardial energy disorder by maintaining mitochondrial homeostasis

**FIGURE 3:**
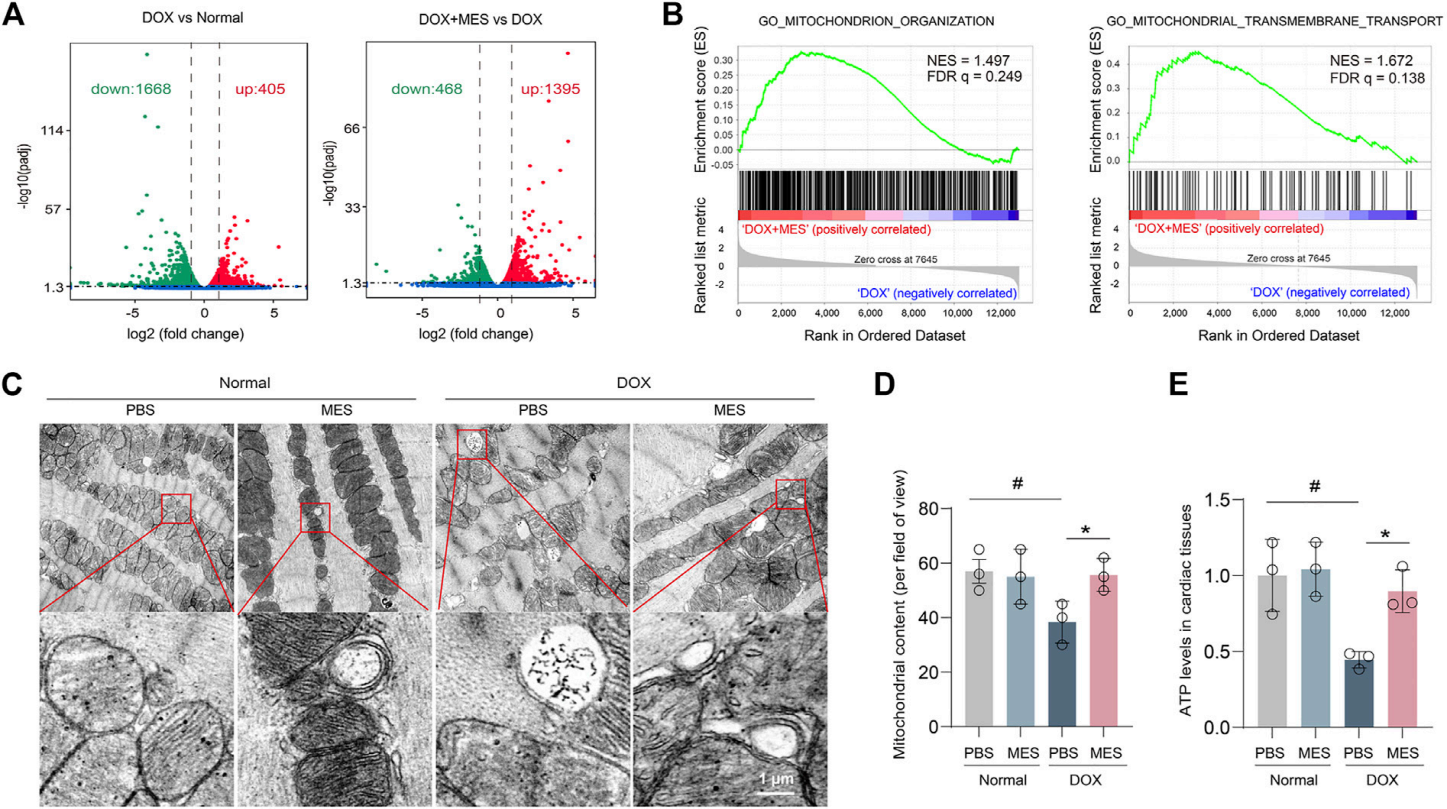
Transcriptome profiles of the myocardium and protective effects of mesaconine on mitochondrial damage. **(A)** Volcano plot of the gene expression values in DOX *versus* normal hearts (left panel) or DOX + mesaconine *versus* DOX hearts (right panel). Red, gene expression upregulated; green, gene expression downregulated in myocardial tissues. **(B)** Gene Set Enrichment Analysis (GSEA) plots of the mitochondrion-related gene modules in the myocardium of mice following the treatment of DOX or DOX and mesaconine. NES, normalized enrichment score; FDR q, false discovery rate q value. **(C)** Representative TEM images of mitochondria (upper panel) and autophagosomes (lower panel) in heart tissues. **(D)** The numbers of mitochondrial contents were determined by quantitative analysis. **(E)** The ATP content in myocardial tissues. Data are represented as means ± SEM of 3 biologically independent replicates assays. *, # *p* < 0.05.

To further explore the mechanism underlying the protective effects of mesaconine in DOX-induced myocardial injury, we examined the gene expression profile of heart tissues. We then found that DOX treatment led to global changes in the mRNA expression of heart tissues compared with that of the control, with a total of 2073 differentially expressed genes identified by RNA-seq. Nevertheless, compared with DOX treatment, there were 1395 genes upregulated and 468 genes downregulated with the cotreatment of DOX plus mesaconine (Figure 3A). More importantly, gene set enrichment analysis of 17,378 genes from heart samples showed that the mitochondria-related gene sets, including their organization and transport, were strongly enriched in the mesaconine and DOX cotreatment group compared with the DOX treatment group (Figure 3B). It is well appreciated that maintaining adequate numbers of functional mitochondria is critical to the overall preservation of cardiac energy supply. We next observed the subcellular structure of heart tissues using transmission electron microscopy (TEM) analysis and found that the cardiomyocytes of DOX-treated mice exhibited abnormal mitochondria with disorganized cristae and markedly reduced mitochondrial content compared to those in healthy mice, which was dramatically ameliorated after mesaconine treatment (Figures 3C, D). As abnormal energy metabolism is the most important consequence and contributing factor in the pathogenesis of heart failure, myocardial ATP levels were examined in each group. Treatment with DOX resulted in a 52% decrease in ATP production in the myocardium, which was completely reversed by mesaconine treatment (Figure 3E). All of these data suggest that mesaconine administration leads to an improvement in energy metabolism by restoring mitochondrial homeostasis in DOX-induced heart failure mice.

### Mesaconine mitigates DOX-induced mitochondrial damage by activating mitophagy

**FIGURE 4:**
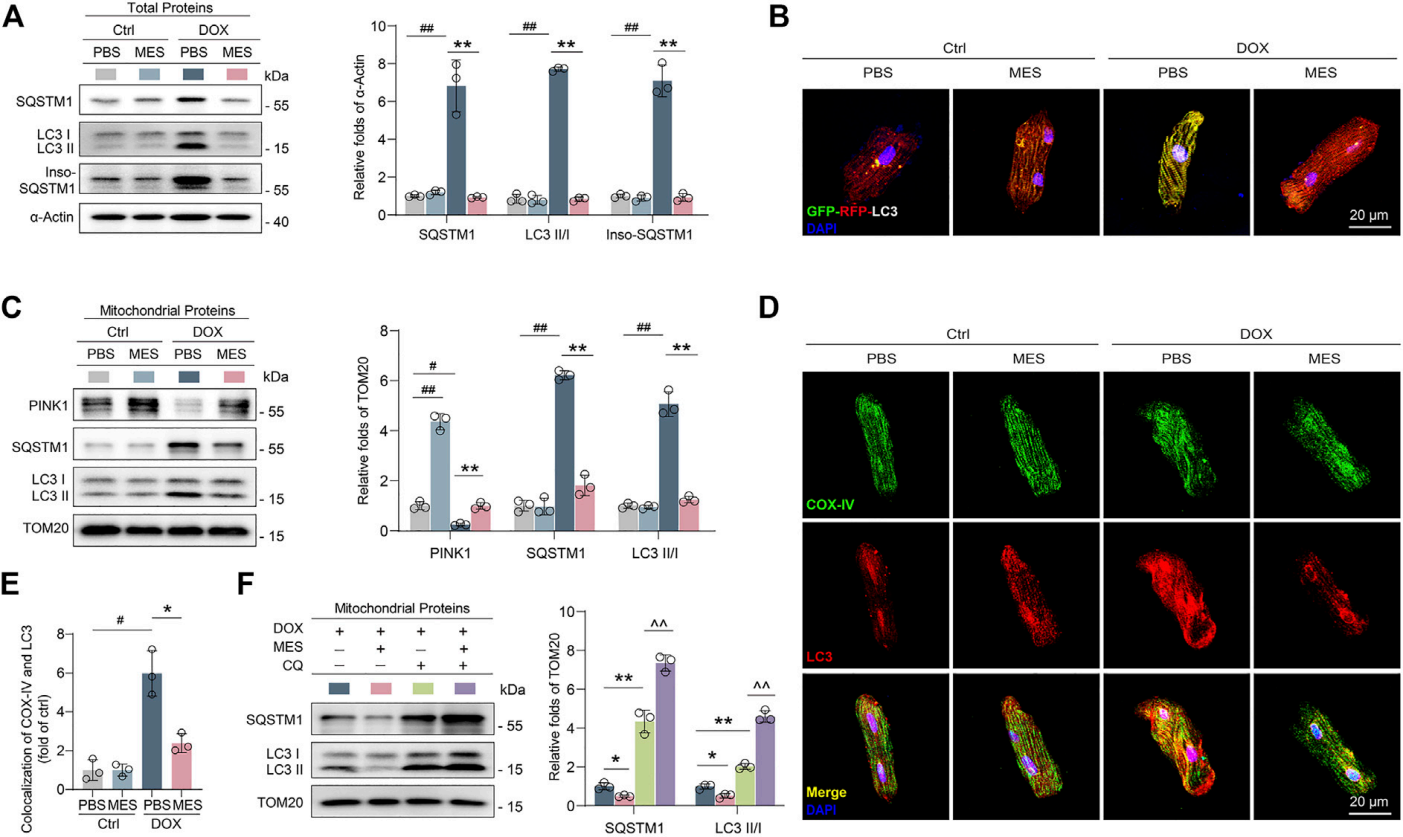
Mesaconine alleviated DOX-induced mitochondrial damage through activating mitophagy. **(A)** Levels of SQSTM1, LC3-II/LC3-I and insoluble SQSTM1 (Inso-SQSTM1) in heart tissues were detected using western blotting (n ≥ 3). **(B)** Cardiomyocytes expressing RFP-GFP-LC3 were treated with DOX and/or mesaconine. Autophagic flux was detected with live-cell imaging microscopy. Representative images showing red-colored autolysosomes or red/green double-colored autophagosomes. **(C)** Levels of PINK1, SQSTM1, and LC3-II/LC3-I in isolated mitochondria form the cardiomyocytes of DOX and/or mesaconine treated mice. **(D, E)** Primary cardiomyocytes were immunostained with an anti-COX-IV Ab and an anti-LC3 Ab to detect the colocalization of autophagosomes with mitochondria. **(F)** Mesaconine-induced restoring of autophagic flux could be turnovered by CQ treatment in mitochondria isolated from the cardiomyocytes of DOX-treated mice. Representative immunoblots and the ratio of the indicated protein to TOM20 are presented. Data are represented as means ± SEM of 3 biologically independent replicates assays. *, # *p* < 0.05; **, ##, ^^ *p* < 0.01.

Given that mitophagy dysfunction is a critical reason for the clearance of impaired mitochondria, it is reasonable to speculate that mesaconine may confer mitochondrial protection *via* targeting potential molecules involved in the processes of mitophagy. Indeed, under an electron microscope, increased autophagosomes (double membrane-bound vacuoles) were obviously visible surrounding the mitochondria in the cardiomyocytes of mesaconine-treated mice (Figure 3C lower panel). To elucidate the potential mitochondrial protection mechanisms of mesaconine, we examined the expression levels of autophagy-associated proteins in isolated rat primary cardiomyocytes and found that DOX treatment not only augmented the expression of SQSTM1 and LC3-II/LC3-I but also resulted in the aggregation of insoluble SQSTM1 in primary cardiomyocytes, indicating obstructed autophagic flux, which could be significantly restored by mesaconine (Figure 4A). To confirm the role of mesaconine in the restoration of autophagic flux, cardiomyocytes were infected with adenovirus expressing the GFP-RFP-LC3 probe to detect autophagic flux based on the concept of lysosomal quenching of GFP. Using live-cell imaging, GFP-RFP-LC3 was observed as red speckles when GFP was degraded by unobstructed autophagy, whereas DOX treatment increased the presence of yellow puncta and dynamically moving yellow spots, indicating autophagy suppression in cardiomyocytes. However, pretreatment of cardiomyocytes with mesaconine restored DOX-suppressed autophagic flux, which was consistent with the data from immunoblotting (Figure 4B). To further elucidate the molecular mechanism by which mesaconine alleviates mitochondrial injury in myocardial tissues, we measured the levels of mitophagy-related proteins, including PINK1, LC3II and SQSTM1, in isolated mitochondria. Consistent with the above data from whole cardiomyocytes, the expression levels of SQSTM1 and LC3-II/LC3-I were increased dramatically following DOX treatment, accompanied by decreased levels of PINK1, a molecular sensor for autophagic degradation of damaged mitochondria. As expected, pretreatment with mesaconine efficiently reversed the disordered expression of SQSTM1, LC3-II/LC3-I and PINK1 (Figure 4C). In addition, mesaconine treatment also significantly reduced the interaction and colocalization of LC3 and COX-IV, a housekeeping marker for mitochondria, in cardiomyocytes treated with DOX (Figures 4D, E). Considering that the mitophagy-restoring effect of mesaconine could be the consequence of either inhibiting the synthesis or promoting the degradation of SQSTM1 and LC3, chloroquine (CQ), a specific inhibitor of late-stage autophagy, was applied in DOX-treated mice to turn over the autophagy-restoring effect of mesaconine. Interestingly, cotreatment with CQ not only enhanced the protein levels of SQSTM1 and LC3-II/LC3-I in myocardial mitochondria from DOX-treated mice but also further increased the expression of these proteins in DOX- and mesaconine-cotreated mice (Figure 4F). Collectively, these data suggest that there is an obstructed autophagic degradation of mitochondria in DOX-injured hearts and that mesaconine treatment activates mitophagy flux to promote the clearance of damaged mitochondria through upregulating the expression of PINK1.

### Depletion of *PINK1* reverses the cardiomyocyte protective effects of mesaconine by inhibiting mitophagy

**FIGURE 5:**
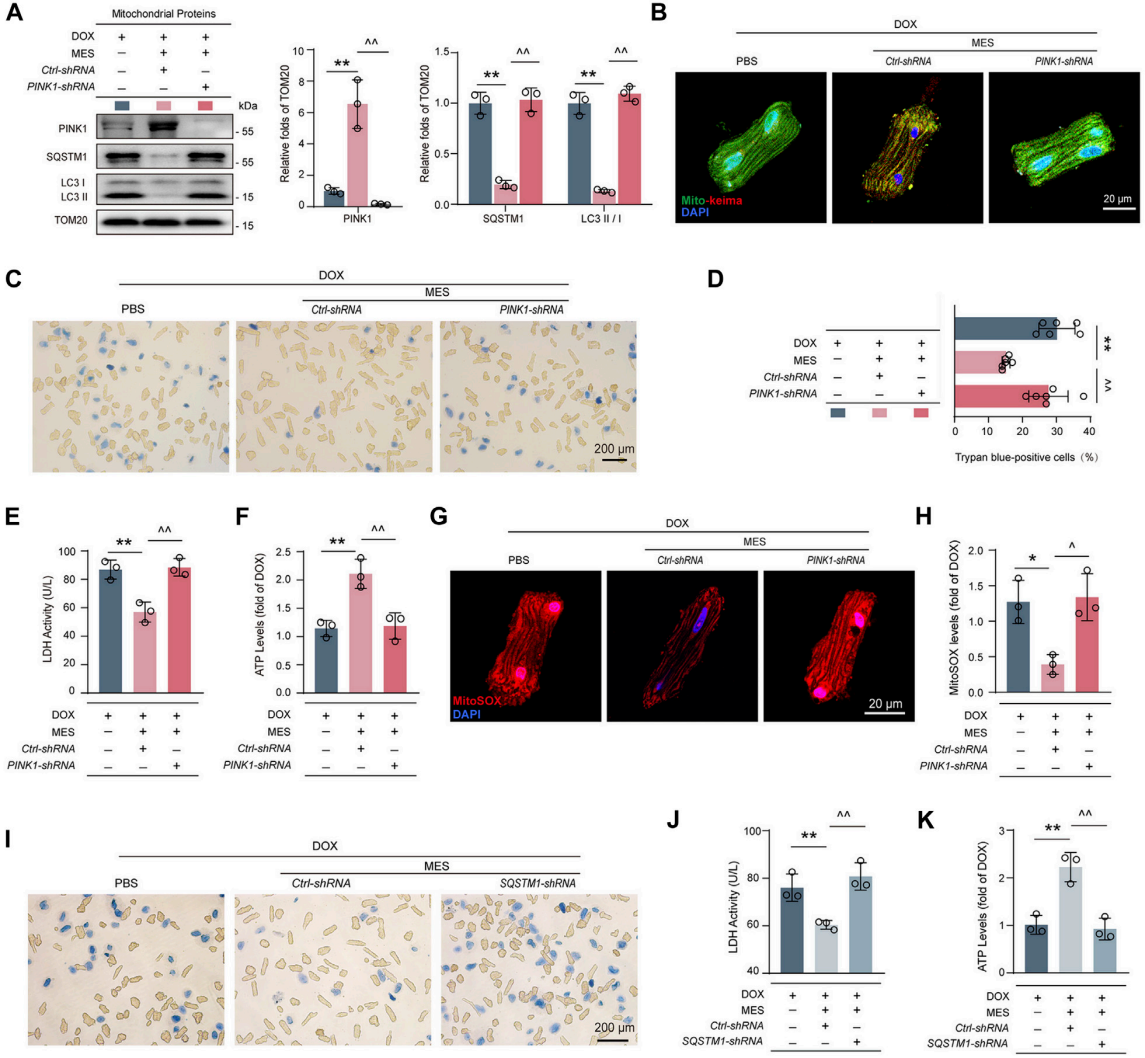
*PINK1* or *SQSTM1* deficiency restricted the cardioprotective effects of mesaconine by inhibiting mitophagy. **(A)** Immunoblotting analysis for the expressions of PINK1, SQSTM1 and LC3II/I in mitochondria isolated primary cardiomyocytes. Representative immunoblots and the ratio of the indicated protein to TOM20 are presented. **(B)** Cardiomyocytes expressing Mito-Keima were used to visualize mitophagy following the treatment of DOX or/and mesaconine under a confocal microscope. **(C)** Representative trypan blue staining images of primary cardiomyocytes. Quantitative analysis of **(D)** trypan blue staining positive cardiomyocytes, **(E)** LDH release, and **(F)** ATP contents of primary cardiomyocytes. **(G)** Representative images of mitochondrial ROS are indicated by MitoSOX red which can be oxidized by superoxide in mitochondria and then produce red fluorescence. **(H)** Quantification of MitoSOX levels. **(I–K)** Trypan blue staining images **(I)** LDH release **(J)** and ATP contents **(K)** of primary cardiomyocytes were assayed and showed that depletion of SQSTM1 reversed the cardioprotective effects of mesaconine. Data are represented as means ± SEM of at least 3 biologically independent replicates assays. *, ^ *p* < 0.05; **, ^^ *p* < 0.01.

To validate whether the pro-mitophagic effect of mesaconine occurred in a PINK1-dependent manner, *PINK1*-depleted cardiomyocytes were treated with DOX and/or mesaconine. We found that knockdown of *PINK1* completely abolished the restorative effect of mesaconine on the expression of mitophagy-related proteins, including SQSTM1 and LC3-II/LC3-I, in mitochondria (Figure 5A). To confirm the role of PINK1 in mesaconine exhibited recovery activity on mitophagic flux, cardiomyocytes were then infected with an adenovirus encoding Mito-Keima to measure mitophagic flux levels. Treatment of cardiomyocytes with mesaconine remarkably restored the DOX-induced obstruction of mitophagy flux, as indicated by decreased green speckles under a confocal microscope, which was completely reversed by *PINK1* depletion (Figure 5B). Based on the findings described above, we wondered whether the protective effects of mesaconine against DOX-induced cardiotoxicity were mediated by regulating PINK1 levels. The cell viability analysis showed that depletion of *PINK1* effectively abrogated the cardioprotective effects of mesaconine on DOX-induced cell death (Figures 5C, D). Similarly, the release of LDH upon DOX treatment, a biochemical marker for necrotic cell death, was significantly suppressed by mesaconine but reversed in *PINK1*-depleted cardiomyocytes (Figure 5E). Additionally, we conducted ATP content assays to evaluate the mitochondrial function and observed that ectopically decreased ATP production in DOX-treated cardiomyocytes was significantly recovered following mesaconine treatment. In contrast, knockdown of endogenous *PINK1* revealed the opposite effects (Figure 5F). Furthermore, upon stimulation with DOX, mesaconine-treated myocytes exhibited a significant decrease in mitochondrial ROS production, as observed from the immunofluorescent staining of MitoSOX. In contrast, depletion of *PINK1* completely deactivated the protective effects of mesaconine (Figures 5G, H). Since PINK1 initiates the ubiquitination of mitochondrial proteins in the outer membrane of damaged mitochondria and SQSTM1 can recognize these ubiquitin-modified substrates and then interact with LC3 to accomplish the encapsulation of mitochondria by autophagosomes, it is reasonable to speculate that SQSTM1 may be involved in the cardioprotection of mesaconine by mediating PINK1-dependent mitophagy. To test this hypothesis, adult rat cardiomyocytes were infected with an adenovirus expressing *SQSTM1*-shRNA to deplete *SQSTM1* before treatment with DOX and/or mesaconine. As expected, depletion of *SQSTM1* completely withdrew the cardioprotective effects of mesaconine, including decreased cell death (Figure 5I) and LDH release (Figure 5J), as well as recovered the ATP content (Figure 5K). These data indicate that mesaconine treatment exerts a potent therapeutic effect against DOX-induced cardiomyocyte injury by activating PINK1-SQSTM1-induced mitophagy, which leads to the timely removal of dysfunctional mitochondria and maintenance of myocardial mitochondrial quality control.

### Mesaconine treatment improves DOX-induced cardiac dysfunction and remodeling in chronic heart failure mice

**FIGURE 6:**
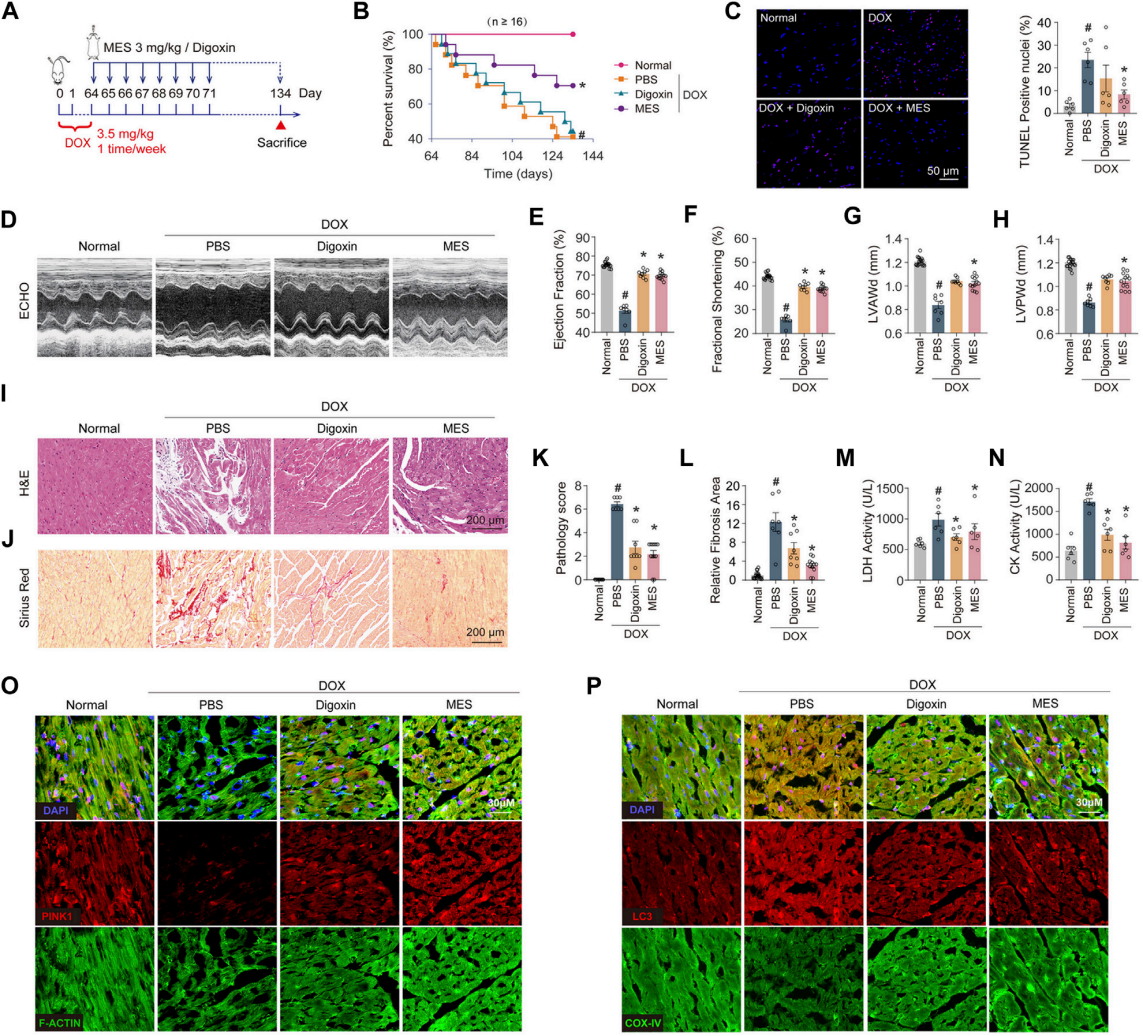
Mesaconine treatment improved left ventricular dysfunction and abnormal myocardial remodeling in DOX-induced chronic heart failure mice. **(A)** The schematic diagram for preparing the heart failure model and corresponding treatments. **(B)** Survival rate curves were analyzed by the Kaplan-Meier method and compared by the log-lank test. **(C)** The TUNEL assay and quantitative analysis in the myocardium. **(D)** Representative images of left ventricular (LV) M-mode echocardiograms. **(E–H)** The LV ejection fraction **(E)**, fractional shortening **(F)**, diastolic anterior wall thickness (LVAWd, **(G)** and posterior wall thickness (LVPWd, **(H)** were measured to evaluate the cardiac systolic function and structure. **(I, J)** Representative images of H&E **(I)** and Sirius Red **(J)** staining of the heart sections. **(K, L)** Quantitative analysis of myocardial histopathological scores **(K)** and collagen deposition **(L)**. **(M, N)** The serum activity of LDH **(M)** and CK **(N)**. **(O)** Levels of PINK1 in myocardial tissues were analyzed by immunostaining. **(P)** Colocalization of LC3 and COX-IV was examined by confocal analysis. Data are represented as means ± SEM (n≥7). *, # *p* < 0.05.

To determine the therapeutic potential of mesaconine for chronic heart failure, we established a chronic mouse model of heart failure induced by multiple injections of DOX for 9 weeks, as shown in Figure 6A. We observed that treatment with mesaconine could markedly improve the survival rate of DOX-induced HF mice in comparison with that of DOX mice treated with digoxin, an anti-HF drug in clinical practice (Figure 6B). Given that accumulated evidence indicates that DOX-induced myocardial injury eventually leads to the apoptotic loss of cardiomyocytes, apoptotic cell death was examined using TUNEL staining in myocardial tissues. The number of TUNEL-positive nuclei was increased by ∼22 times in the myocardium upon DOX treatment relative to normal hearts. Whereas mesaconine treatment significantly alleviated DOX-induced cardiomyocyte apoptosis (Figure 6C). Furthermore, echocardiography was performed to evaluate cardiac function and morphology. Mesaconine exerted a functional and constructional improvement in HF (Figure 6D), as indicated by increased LV ejection fraction (Figure 6E) and fraction shortening (Figure 6F), as well as elevated LV end-diastolic anterior wall thickness (Figure 6G) and posterior wall thickness (Figure 6H). Next, we sought to investigate whether mesaconine treatment mitigates DOX-triggered cardiac remodeling and fibrosis. We found that DOX-induced cardiotoxic injury resulted in extensive loss of cardiomyocytes and myocardial fibrosis, manifested as strikingly increased intercellular space enlargement and collagen accumulation, which could be remarkably attenuated by treatment with either mesaconine or digoxin (Figures 6I, J). In addition, mesaconine treatment resulted in a decrease in histopathology score and fibrosis area of myocardial tissues compared with model mice (Figures 6K, L), accompanied by reduced LDH (Figure 6M) and CK activity (Figure 6N) in serum. Moreover, confocal microscopy analysis revealed that cardiac tissues exhibited decreased levels of PINK1 in cardiomyocytes, as well as enhanced colocalization of LC3 with COX-IV, which could be markedly reversed by mesaconine treatment (Figures 6O, P). Taken together, these data confirm that mesaconine exerts a potent therapeutic effect against cardiac dysfunction and remodeling in DOX-induced heart failure mice through targeting PINK1-dependent autophagic clearance of damaged mitochondria.

## Discussion

**FIGURE 7:**
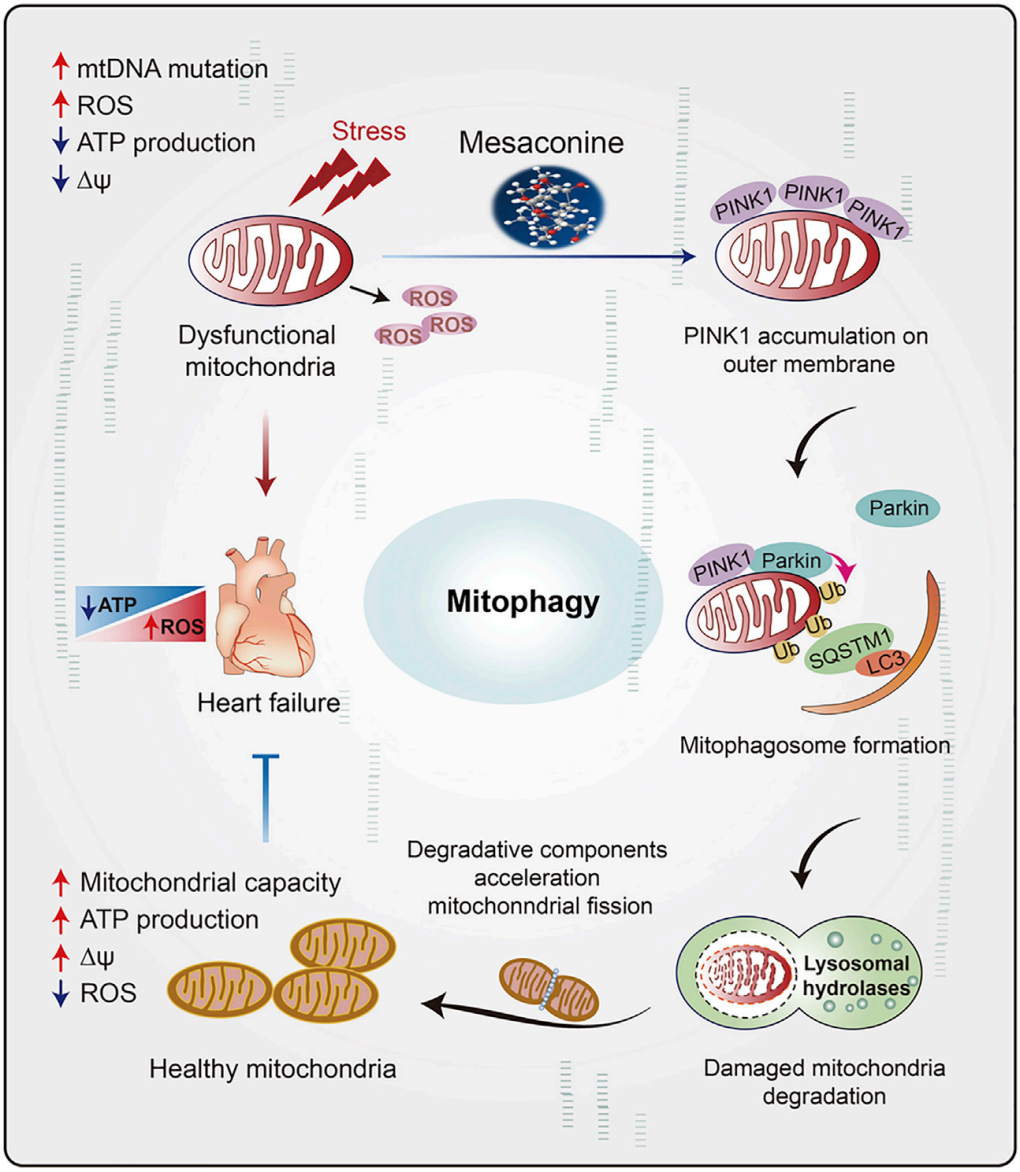
The schematic diagram illustrates the role of mesaconine in restoring energy metabolism disorder in the myocardium through activating PINK1-SQSTM1-mediated mitophagy, which makes mesaconine an attractive therapeutic agent for the treatment of heart failure and other diseases of myocardial energy metabolism.

The clinical application of DOX is severely hampered by its dose-dependent cardiotoxicity and consequent heart failure (Angsutararux et al., 2015), characterized by ROS overproduction, imbalance of mitochondrial dynamics (Li et al., 2022), cardiac energy metabolism dysfunction and cardiomyocyte apoptosis (Sterba et al., 2013). Dexrazoxane (DXZ) is currently the exclusive FDA approved drug for DOX induced cardiotoxicity treatment. However, the clinical application of DXZ is restricted because it might also protect the cancer cells against chemotherapy (Chen et al., 2022). Numerous natural compounds, such as resveratrol and ginsenosides, have been tested for anti-DOX-induced cardiomyopathy in clinical trials, but few of them could genuinely achieve a clinical needs assessment (Tatlidede et al., 2009; Liu et al., 2020). Mesaconine, a natural diterpenoid alkaloid compound isolated from the lateral roots of *Aconitum carmichaelii*, is the main cardiotonic component in “Fuzi” (Jian et al., 2012). It has been reported that the single maximum tolerated dose of mesaconine in rats reached 1200 mg/kg, leading to no appreciable toxicity or adverse reactions, which implies its safety as a novel anti-heart failure medicine (Wang and Chao, 2021). However, the precise molecular mechanism and target of mesaconine remain elusive. Herein, our data demonstrated for the first time that treatment with mesaconine could significantly ameliorate cardiac dysfunction and mitochondrial damage induced by DOX, particularly with a marked reduction in mitochondrial ROS and myocardial energy disorder, through restoring PINK1-mediated mitophagy (Figure 7). In the present study, we not only revealed a previously unidentified cardioprotective mechanism for mesaconine but also suggested an attractive therapeutic candidate for treating heart disease with energy metabolism disorder.

Maintaining healthy and functional mitochondrial pools has paramount importance in producing an enormous amount of ATP to meet the high energy demand of the heart (Lopaschuk et al., 2021). Accumulated evidence indicates that DOX-induced cardiotoxicity is mainly associated with dysregulated ROS generation (Gorini et al., 2018). Mitochondria are the primary source of ROS in the heart under pathological conditions. Excessive production of mitochondrial ROS results in increased damage to mtDNA, accumulation of mitochondrial superoxide, decreased ATP production and potential depolarization of the mitochondrial membrane (Wang et al., 2016). Therefore, maintaining mitochondrial homeostasis might be an effective strategy for cardioprotection. Our RNA sequencing analysis implies that the therapeutic effect of mesaconine on DOX-induced cardiomyopathy is remarkably correlated with energy metabolism and mitochondrial signaling pathways. Indeed, mesaconine treatment almost completely reversed the DOX-induced decrease in ATP production and elevated mitochondrial ROS in cardiomyocytes.

Damaged mitochondria, which are detrimental, are usually eliminated by mitophagy if the intracellular mitophagic machinery works properly (Saito et al., 2021). Alternatively, the obstructed mitophagy pathway will fail to clear damaged or depolarized mitochondria, which results in excessive ROS release and even cardiomyocyte death (Sciarretta et al., 2018; Wang and Zhou, 2020). We recently reported that dysregulation of lysosomal autophagy is involved in the pathogenesis of cardiomyopathy induced by DOX in a TLR2-dependent manner and that blocking TLR2 activity provides therapeutic benefits in mouse models of heart failure (Ma et al., 2012). Additionally, exposure to DOX can significantly suppress mitophagy and block the clearance of injured mitochondria in cardiomyocytes by inhibiting Parkin, an E3 ubiquitin ligase that collaborates with PINK1 to initiate mitophagy (Li et al., 2022b). Our study further demonstrated an aberrant mitophagic flux in DOX-injured hearts and cardiomyocytes, as observed by the reduction in PINK1 protein levels and ectopic aggregation of SQSTM1 and LC3, as well as the accumulation of damaged mitochondria.

PINK1-mediated mitophagy is the most comprehensively characterized mechanism for mitochondrial quality control that targets impaired mitochondria and degrades them to maintain mitochondrial homeostasis (Zhou et al., 2020). PINK1 is a serine/threonine-protein kinase that is constitutively located at the inner mitochondrial membrane (IMM) and degraded by mitochondrial protease in healthy mitochondria (Mukherjee et al., 2015). Non-etheless, mitochondrial damage or depolarization will facilitate PINK1 accumulation at the outer mitochondrial membrane (OMM) and recruit Parkin to promote large-scale ubiquitination of OMM proteins (Gegg et al., 2010). Polyubiquitinated mitochondria can be recognized by Ub-binding receptors such as SQSTM1, captured by autophagosomes and then delivered into lysosomes, in which resident hydrolases degraded ubiquitinated mitochondria (Lazarou et al., 2015; Dikic, 2017). Since the consumption of PINK1 in the process of mitophagy is non-recyclable, once the protein level of PINK1 is decreased under pathological conditions, impaired mitophagy would cause the accumulation of damaged mitochondria and further cell injury. Indeed, it has been demonstrated that PINK1 protein levels are significantly reduced in human end-stage heart failure and that PINK1 activity is essential for correct postnatal myocardial development (Billia et al., 2011), suggesting that mitophagy impairment contributes to the pathological process of heart disease. PINK1 is important for maintaining healthy heart function. Consistent with previous findings, our present study verifies that DOX-induced cardiotoxicity is associated with PINK1-mediated mitophagy. More importantly, mitophagy-related components, including LC3 and SQSTM1 and their insoluble form, are extremely aggregated in DOX-treated cardiomyocytes, indicating that mitoautophagic flux is impaired and lacks sufficient capacity to deal with damaged mitochondria. Here, we determined that the cardioprotective effect of mesaconine is mainly related to restoring PINK1 levels in mitochondria.

In conclusion, we showed here that mesaconine-targeted PINK1 confers a cardiomyocyte protective effect against DOX-induced myocardial energy disorder by restoring mitochondrial homeostasis. The underlying mechanism is associated with the activation of SQSTM1-mediated mitophagy. Our current findings from both *in vivo* and *in vitro* studies suggest that mitochondrial dysfunction is an essential signature of DOX-induced myocardial injury and that mesaconine may serve as a novel candidate compound targeting mitochondrial homeostasis to fight against DOX-induced cardiomyopathy, and other cardiac diseases caused by mitochondrial oxidative stress. From a clinical perspective, *Aconitum carmichaelii* has been widely used as a cardiotonic drug in the clinical applications of traditional Chinese medicine (Liu et al., 2012; Yang et al., 2014; Chen et al., 2022). Nowdays, the Shenfu Injection is still a successful traditional Chinese medicine formulation proved with both efficacy and safety (Tao et al., 2023). Our present study not only demonstrate that the *Aconitum carmichaelii* extracted cardiotonic component Mesaconine could significantly improve the heart function with a wide therapeutic window, but also illustrate that the PINK1 dependent mitophagy was involved in the mesaconine’s cardio-protection effects. These findings have major implications for the design of clinical trials of mesaconine in anti-HF development pipeline. However, further studies are needed to fully elucidate the pharmacological consequences of mesaconine and gain insight into its molecular mechanisms. First, the specific interacting proteins of mesaconine in the regulation of the mitophagy pathway remain unclear. Additionally, it is largely unknown whether other mitophagy receptors, such as BNIP3, FUNDC1, and SMURF1, are involved in the mesaconine-mediated regulation of mitophagy (Liu et al., 2014; Palikaras et al., 2018).

## Materials and methods

### Isolation of primary adult rat cardiomyocytes

Cardiomyocytes were isolated with a modified langendorff perfusion system. Briefly, rat was anaesthetized by intraperitoneal injection with 1.25% tribromoethyl aicolaol (Sigma, T48402) and 5000 unit/kg heparin (Solarbio, H8060) were injected intraperitoneally to prevent blood clotting. Then the heart was removed out together with about 1.5 cm of aortic arch. Immediately afterwards, the heart was perfused from the aortic with KHB buffer (118 mM NaCl, 4.8 mM KCl, 25 mM HEPES, 0.6 mM KH_2_PO_4_, 1.25 mM MgSO_4_, 11 mM Glucose, 5 mM Taurine, 10 mM BDM, pH = 7.4), and then digested the heart for 25 min with solution E buffer, which contained with 1 mg/ml BSA (Sigma, V900933), 0.7 mg/ml collagenase-II (Worthington-Biochem, LS004176), 0.2 mg/ml hyaluronidase (Sigma, H3506) and 25 μM CaCl_2_. After perfusion, the heart was gently minced in a sterile dish and then filtered with a 100 μm cell strainer. Cardiomyocytes suspension was separated twice with solution B buffer (10 mg/ml BSA, 0.1 mM CaCl_2_) by gravity sedimentation, then the primary cardiomyocytes were seeded in the laminin (Gibico, 23017-015) coated plate for further experimentation.

### Cardiomyocytes culture and pharmacological treatments

Primary cardiomyocytes were cultured in M199 medium (Gibco,11150059) containing 10% fetal bovine serum (Viva cell, C04001500), 1% penicillin-streptomycin (Thermo Fisher, 15070063) and 1% glutamine (Gibco, 35050061) at 37°C, 5% CO_2_. Cardiomyocytes were stimulated with doxorubicin (DOX, J&K Scientific, 113424) and mesaconine (Sichuan Engineering Research Center for Medicinal Animals, Sichuan, China). The compound mesaconine, formulated as (3R, 4R, 6S, 7R, 7aR, 9R, 10S, 11S, 11aR, 13R, 14S)-6, 10, 13-trimethoxy-3-(methoxymethyl)-1-methyldodecahydro-11aH-3,6a,12-(epiethane [1,1,2]triyl)-7,9-methanonaphtho [2,3-b]azocine-4,8,9, 11,11a (7H)-pentaol (Figure 1A), was dissolved in phosphate buffered saline for administration. The CCK-8 assay was performed to evaluate cardiomyocytes viability after DOX and mesaconine treatment. Briefly, cardiomyocytes were seeded in 96 well plate at a number of 5000 per well. Then cardiomyocytes were treated with DOX (8.6 μM) and different concentrations (0, 0.8, 4, 20, 100, 500, 2500 μM) mesaconine for 24 h to evaluate the viability with CCK-8 assay kit (DOJINDO, CK18). Then the cardiomyocytes were seeded in 6 well plates and treated with or without indicated agents. The LDH release was measured using biochemical detector. The ATP level of cardiomyocytes was measured using ATP assay kit (Beyotime, S0027) according to the manufacturer’s instructions. The samples or standard ATP solution were mixed with ATP detection working solution and incubated for 5 min, then the RLU was determined with a microplate reader (Biotek, Synergy H1), cardiomyocytes cytoplasma protein concentrations of each group were determined for the further normalization of ATP content. The trypan blue staining assay (Solarbio, T8070) was used to measure cardiomyocytes viability, and the trypan blue stained cardiomyocytes were viewed with a microscope (OLYMPUS, BX51) and the percentage of positive cells were calculated.

### Animal model and treatments

ICR mice (male, 8-week-old, SPF grade, Certification No. 11401500027366) were purchased from Sibef Biotechnology Co. Ltd. (Beijing). All animal studies were approved by the Animal Experimentation Ethics Committee of the Chinese Academy of Medical Sciences, and all procedures were conducted following with the guidelines of the Institutional Animal Care and Use Committees of the Chinese Academy of Medical Sciences.

To examine the effect of mesaconine on DOX-induced acute cardiotoxicity, mice were randomly divided into four groups: Normal, mesaconine, DOX and DOX + mesaconine. Acute cardiotoxicity was induced in ICR mice by a single intraperitoneal (i. p.) injection of DOX at a dose of 15 mg/kg over a period of 2 weeks, whereas control mice received equal volume of saline. And then mice were treated with mesaconine or saline every day for 2 weeks (Figure 2A). At day 28 after DOX injection, LV structural and functional changes were evaluated by echocardiography.

Chronic heart failure was induced in ICR mice by i. p. injection of DOX (3.5 mg/kg) weekly for 9 weeks, whereas control mice received equal volume of saline. At day 63 after the initial DOX injection, the mice were randomly divided into the 3 groups and treated with digoxin, mesaconine or saline for additional 10 weeks (Figure 6A). At the experimental endpoint, LV structural and functional changes were evaluated by echocardiography.

At the end of experiment, the mice were sacrificed by excessive anesthesia for collection of serum and hearts.

### Echocardiography

The data of echocardiography was obtained with the Vevo 770 High-Resolution Micro-Imaging System (VisualSonics, Canada) using a 30-MHz image transducer. At first, preparing the chest skin of the experimental mice and anesthetized the mice with 1.25% tribromoethyl aicolaol. Proceed the Echo under the mice physiology condition of ∼500 beats per min heart rate to guarantee the accuracy of cardiac function measurement. Then, the mice were fixed on the operating table (37°C) in a supine position. Two-dimensional image was obtained and collected parameters include left ventricular ejection fraction (EF), left ventricular fractional shortening (FS), left ventricular end diastolic anterior wall thickness (LVAWd), and left ventricular end diastolic posterior wall dimension (LVPWd).

### CK and LDH activity assay

Mice blood was collected and centrifuged at 4500 rpm for 15 min at 4°C to obtain serum. The CK and LDH activity of mice serum were used to evaluate the myocardial injury, and the CK and LDH activity of mice serum were assayed by biochemical detector.

### Morphological and histological evaluation

Mouse hearts were fixed with 4% paraformaldehyde (Bioss, C0106002) and embedded with paraffin, and sectioned at 5 μm thickness. Hematoxylin and eosin (H&E) or Sirius red were used to strain the cardiac tissue sections according to standard protocols. The sections were viewed under a microscope to evaluate the pathology score or relative fibrosis area, which were blindly assessed by an experienced pathologist.

### Immunofluorescence staining

Frozen sections of heart tissue or primary cardiomyocytes were fixed with 4% paraformaldehyde for 10 min, after permeabilized with 0.2% Triton X-100, samples were subsequently blocked with 3% BSA for 60 min, which were then incubated overnight at 4°C with the following primary antibodies: anti-LC3B (1:100, Sigma, L7543); Anti-COX-IV (1:100, Abcam, ab33985). The samples were stained with Alexa Fluor 488- or Alexa Fluor 647-conjugated secondary antibodies for 2 h in dark at room temperature. Finally, mounting medium with DAPI reagent (ZSGB-BIO, ZLI-9557) was used to seal the slides, and images were acquired using a fluorescence microscope.

### Apoptosis analysis

DNA fragmentation of apoptotic cells was analyzed by TUNEL staining detection kit (Roche, 11684817910) and counterstained with DAPI. Images were acquired by a confocal microscope (OLYMPUS, FV3000). Then the average percentage of apoptosis was calculated.

### Transmission electron microscopy

Heart tissues (1 mm^3^) were fixed overnight in triple buffer (pH 7.4) containing 2.5% (w/v) glutaraldehyde, 0.01% picric acid and 0.1 M cacodylate buffer. Rinsed the heart tissues in the phosphate buffer (0.1 M, pH 7.4) and then immersed in 1% (v/v) osmium tetroxide for 1 h followed by block incubation with 2% (v/v) aqueous uranyl acetate for 2 h. After dehydrated tissues with a graded series of ethanol, the samples were washed with acetone and embedded in araldite. Then the sample was cut at least three pieces of ultrathin sections (75–80 nm), collected on 200-mesh copper grids and stained with 5% uranyl acetate in ethanol (10 min) and lead citrate (5 min). The grids were examined by transmission electron microscopy (Hitachi H600, Japan) operated at 80 KV. Random fields of view under the same magnification were chosen form per sample and the number of mitochondria within each field was counted.

### RNA isolation and quantitative real-time PCR

Total RNA was extracted from mouse hearts with Trizol reagent (Sigma, T9424), after determined concentrations, 1 µg RNA was reverse transcribed into cDNA using iScript cDNA reverse kit (BIO-RAD, 1708891) with the total volume of 20 µl according the standard protocol. Real-time PCR was performed using universal Blue qPCR SYBR Green Master Mix (Yeasen, 11184ES03). The primer sequences were presented following: *ANP*-F: 5′-ATC TGC CCT CTT GAA AAG CA-3′; *ANP*-R: 5′-ACA CAC CAC AAG GGC TTA GG-3′; *BNP*-F: 5′-CAG CTC TTG AAG GAC CAA GG-3′; *BNP*-R: 5′-AGA CCC AGG CAG AGT CAG AA-3′; *α-Actin*-F: 5′-TCC AGC CAT CTT TCA TTG GGA-3′; *α-Actin*-R: 5′-CCC CTG ACA GGA CGT TGT TA-3′.

### Transcriptome sequencing

Mice heart tissues from different groups (n = 3 per group) were used for RNA-seq. RNA library preparation, sequencing, and analysis were performed by Orvisen Gene Technology Ltd. (Beijing). Briefly, RNA of heart tissues was isolated with Trizol, and transcriptome sequencing libraries were constructed and sequenced on the second-generation high-throughput illumina platform. After quality control, STAR and Cufflinks software were used to perform the transcriptome sequencing and data analysis, and the fragments per kilobase of transcript sequence per million (FPKM) data were used for further analysis.

Gene set enrichment analysis (GSEA) was performed with GSEA 4.2.3. We ranked the 17,378 genes by their association with DOX (n = 3) and DOX + mesaconine (n = 3) group, using the signal-to-noise measure in the gene-set enrichment analysis (GSEA). Differentially expressed genes (DEGs) were identified using the DESeq2 package (fold change ≥1.2, *p* < 0.05) (Subramanian et al., 2005).

### Mitophagy flux assay and adenoviruses infection

For the mitophagy detection *in vivo*, mice heart tissues homogenates were centrifugated at 4°C, 12000 rpm for 30 min, the supernatant protein concentrations were determined by BCA. Then, the insoluble pellets were washed 3 times with lysis buffer and resuspended in 2% SDS in Tris buffered saline (40 mM Tris, 8 M urea, 4% CHAPS) with an adjusted volume according to the protein concentration in the supernatant to guarantee equal amounts of proteins were subject to SDS-PAGE.

Primary cardiomyocytes were plated on small confocal dishes, and then were infected with Mito-Keima, adenoviruses encoding *Ctrl*-shRNA or *PINK1*-shRNA (Target Seq: GGG​TTC​AGC​AAA​CAG​GAA​G, Dose: 1 × 10^8^ PFU/ml). After 24 h, the medium was discarded and replaced with fresh medium containing DOX or mesaconine treatment for 6 h. Finally, cardiomyocytes were observed under confocal microscope (OLYMPUS, FV3000). The different pH of autophagosome (neutral) and autolysosome (acidic) allows us to monitoring the mitophagy flux by fusing a tandem repeat of the COX VIII presequence to mKeima. Under laser scanning confocal microscope, the neutral condition (pH = 7) mitochondria reflect fluorescence signals at 440 nm while the mitochondria in autolysosome (pH = 4) reflect fluorescence signals at 586 nm.

### Measurement of mitochondrial ROS

Primary cardiomyocytes were plated in small confocal dishes. Then, cardiomyocytes were incubated with DOX or mesaconine for 6 h. Five mM MitoSOX^TM^ reagent (Thermo fisher, M36008) stock solution was diluted with HBSS buffer to make a 5 μM working solution buffer. Cardiomyocytes were then incubated with 1 ml working solution buffer at 37°C for 10 min in dark according to the manufacturer’s instructions. Images were captured using a fluorescence microscope.

### Isolation of mitochondrial protein

Mitochondrial proteins were isolated with Minute^TM^ Mitochondria Isolation Kit (Invent, MP-007) according to the manufacturer’s instructions. Fresh heart tissue (20–30 mg) was placed in a filter cartridge, and incubated with 250 µl buffer A for 5 min at 4°C. After centrifuged 16,000 G for 30 s, precipitation was resuspended by vortexing briefly. Subsequently centrifuged at 700 G for 1 min, supernatant was transferred to another 2 ml tube and added 400 µl buffer B mixing by vortexing for 10 s. After series of centrifugation, mitochondrial protein was obtained for further using.

### Immunoblot analysis

Heart tissues or cells were collected and lysed with RIPA buffer (Beyotime, P0013C) containing protease inhibitors (Selleck, B15002). The samples (20 µl) were used to determine the total protein concentrations with BCA reagent kit (Applygen, P1511-1). Protein samples were heat-denatured for 5 min at 95°C with the sample loading buffer, and 20 µg of total protein per sample were separated by SDS-PAGE gels and transferred to PVDF membranes (Millipore, IPVH00010). The membranes were blocked with 5% BSA for 30 min and incubated overnight at 4°C shaking with primary antibodies. The membranes were then washed in Tris-buffered saline (TBS, pH 7.6) with 0.1% Tween-20 and incubated with horseradish peroxidase-conjugated secondary antibodies (1:4000, ZSGB-BIO, ZB2301) for 2 h at room temperature. Bands were visualized using ECL Detection Reagent (Applygen, P1050), and quantify analysis was performed using Gel-Pro Analyzer System. The primary antibodies used were presented as follow: anti-LC3B (1:5000, Sigma, L7543); anti-p62/SQSTM1 (1:5000, Sigma, P0067); anti-PINK1 (1:1000, Abcam, ab23707); anti-TOM20 (1:1000, Abcam, ab78547); anti-α-Actin (1:5000, Abcam, ab156302).

### Statistical analysis

All data are represented as mean ± SEM. Multiple groups comparisons among three or more groups were conducted using one-way ANOVA, in which Shapiro-Wilk test was performed for normality. Thereafter, Dunnett’s T3 multiple comparisons tests were performed with assessment of statistical significance. Survival curves were obtained using the Kaplan-Meier method and compared by the log-lank test. A value of *p* <0.05 or *p* <0.01 was considered statistically significant. All statistical results were analyzed using Graphpad Prism 9.0 software.

## Data Availability

The original contributions presented in the study are included in the Supplementary Material. The RNA-seq data are deposited in the Sequence Read Archive: https://www.ncbi.nlm.nih.gov/sra under the accession number PRJNA949273. Further inquiries can be directed to the corresponding author.
